# The 2012 Madeira Dengue Outbreak: Epidemiological Determinants and Future Epidemic Potential

**DOI:** 10.1371/journal.pntd.0003083

**Published:** 2014-08-21

**Authors:** José Lourenço, Mario Recker

**Affiliations:** 1 Medical Research Council Centre for Outbreak Analysis and Modelling, Department of Infectious Disease Epidemiology, School of Public Health, Imperial College London, London, United Kingdom; 2 Department of Zoology, University of Oxford, Oxford, United Kingdom; 3 College of Engineering, Mathematics & Physical Sciences, University of Exeter, Penryn Campus, Penryn, United Kingdom; The University of Texas at Austin, United States of America

## Abstract

Dengue, a vector-borne viral disease of increasing global importance, is classically associated with tropical and sub-tropical regions around the world. Urbanisation, globalisation and climate trends, however, are facilitating the geographic spread of its mosquito vectors, thereby increasing the risk of the virus establishing itself in previously unaffected areas and causing large-scale epidemics. On 3 October 2012, two autochthonous dengue infections were reported within the Autonomous Region of Madeira, Portugal. During the following seven months, this first ‘European’ dengue outbreak caused more than 2000 local cases and 81 exported cases to mainland Europe. Here, using an ento-epidemiological mathematical framework, we estimate that the introduction of dengue to Madeira occurred around a month before the first official cases, during the period of maximum influx of airline travel, and that the naturally declining temperatures of autumn were the determining factor for the outbreak's demise in early December 2012. Using key estimates, together with local climate data, we further propose that there is little support for dengue endemicity on this island, but a high potential for future epidemic outbreaks when seeded between May and August—a period when detection of imported cases is crucial for Madeira's public health planning.

## Author Summary

In 2012, Europe saw its first dengue epidemic taking place on the Atlantic island of Madeira. Due to strong tourism links, 81 cases were introduced into continental Europe in a short period of three months. Although *Aedes aegypti*, the mosquito-vector responsible for this particular outbreak, is extinct in mainland Europe, climatic and globalization trends have eased the recent establishment of *Aedes albopictus*, dengue's secondary vector, in France, Germany, Italy and Spain. Before this epidemic, dengue had only sporadically achieved short chains of transmission. The presence of fully susceptible populations, however, makes the possible introduction into Europe a major public health concern. Here, using a mathematical approach, we analysed Madeira's dengue outbreak, focusing on the necessary conditions for introduction, spread and persistence. We find that natural temperature cycles were the determining factor for the 2012's outbreak demise, and are generally expected to severely disrupt dengue transmission between November and April, suggesting weak potential for endemicity. On the other hand, Madeira demonstrates a high potential for sporadic and potentially large epidemics in the remaining summer months, especially if the virus is introduced early during the warm season.

## Introduction

The ongoing spread of dengue, the most important mosquito-borne flavivirus affecting humans, from predominantly tropical and sub-tropical regions into higher latitudes, such as the United States of America, Australia and Europe, is a major public health concern [1]. Globalisation and climate change are some of the possible factors that have facilitated the geographic expansion of its two vector-species, *Aedes aegypti* and *Aedes albopictus* [2], [3]. The size of the dengue-naive population together with frequent travels to endemic countries impose a significant risk of large epidemic outbreaks in these regions as well as the possibility of dengue becoming (re-)established as an endemic disease [4]. Understanding and quantifying the potential of dengue outbreaks in previously dengue-free environments is therefore paramount for public health planning.

*Aedes aegypti*, dengue's main vector, has been considered extinct from continental Europe since the mid-twentieth century but was recently introduced to the The Portuguese Autonomous Region of Madeira [5]. This Atlantic archipelago consists of several islands, two of which are inhabited. From these, the island of Madeira is the largest with a population size of 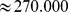. It has an approximate area of 750 square kilometres and is located around 1000 kilometres from the European continent, sharing roughly the same latitude as central Morocco. The interior of Madeira is particularly mountainous, which has resulted in its population being distributed mainly along the coast, specially in the south, where the capital city of Funchal, harbouring nearly half of the island's inhabitants, is located.

The mixture of densely populated areas with rich and abundant sub-tropical vegetation will have promoted the mosquito's introduction into Funchal, from where it spread longitudinally along the coast and later to the rest of the island [5]. In contrast to many dengue-endemic cities in tropical regions, mosquito breeding in Funchal can not be linked to poor sanitation, waste disposal or water storage practices [6], [7]. Instead, the well established habit of potting small plants and flowers provides a vast number of potential breeding sites, both indoors and surrounding domestic premises [5].

**Figure 1:**
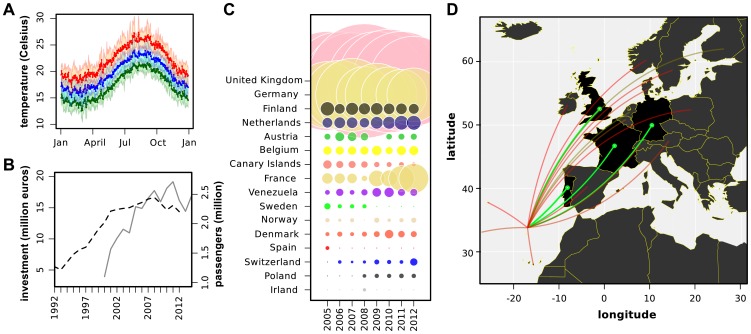
Tourism and temperature data for the island of Madeira. (*A*) Mean of minimum (green), average (blue) and maximum (red) temperatures per day between 2002 and 2012. Coloured areas are the standard deviation. (*B*) Number of airline passengers entering Madeira per year (dashed, black) and local investment in tourism per year (solid, grey). (*C*) Relative weight (bubbles) of each country in the total number of passengers arriving at Madeira per year (columns). Data compiled from the 30 most frequent cities of origin for airline passengers per year. Portuguese cities were excluded - Oporto, Lisbon, Porto Santo (Madeira) and Ponta Delgada (Azores). (*D*) Map representation of (C), including Portugal. Colours match the weight of each country with the 4 highest highlighted in green.

Although Madeira's climate is classified as Mediterranean, its heterogenous landscape imposes significant differences in sun exposure, humidity and mean daily temperatures. These local variations, together with influences from the Gulf Stream and the Canary Current, develop into a range of contrasting local microclimates. The island presents monthly average temperatures above 20° Celsius during spring, summer and autumn, peaking around 26° Celsius in August (Figure 1A). Even during the winter months, temperatures often remain above 15° Celsius. The mild climate together with the blend of seaside, mountainous and urban landscapes, and short flight distances to continental Europe, make the island of Madeira an attractive tourist destination. In the past two decades, successive governments have successfully invested in the expansion of the tourism industry, transforming it into the main driving force of the small, local economy. Consequently, the Archipelago has witnessed a major increase in the number of international airline travellers (Figure 1B), mainly from Europe but also from South America (Figures 1C and D).

**Figure 2:**
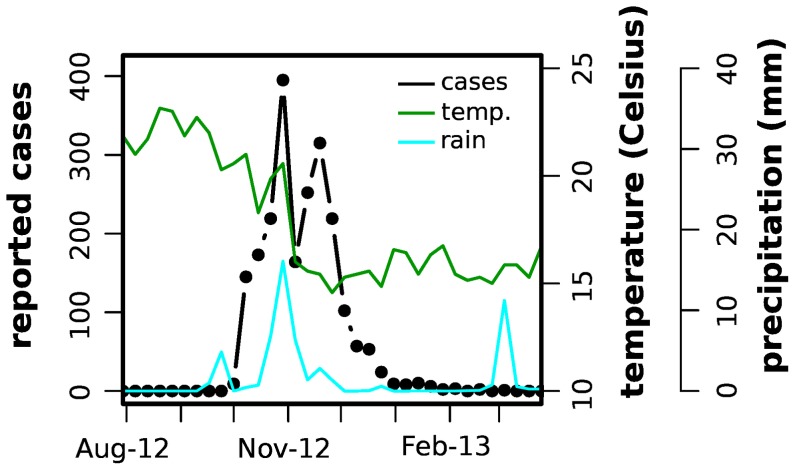
Climate and dengue outbreak data for the island of Madeira. Mean of minimum temperatures per week (solid, green), precipitation (solid, cyan) and dengue reported cases per week (dotted, black) for August-2012 to March-2013.

On 3 October 2012, two dengue infections were reported by the Direcção Geral de Saúde (Portuguese health ministry) on the island of Madeira [8], [9]. The patients had no recent overseas travel history, raising an alert for possible autochthonous transmission. In the following weeks the island witnessed its first dengue epidemic with a total of 2187 reported cases, of which approximately 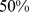 were confirmed [9]. The outbreak was characterized by a sharp increase in weekly reported cases throughout October, peaking in November and decreasing rapidly thereafter (Figure 2). It was declared extinct on March 2013, after which one case was imported from Brazil and two others from Angola (until the end of summer 2013) [9], [10]. During the epidemic period, 81 cases were exported to continental Europe, with 11 reported cases in Portugal and 70 in other European countries [9]. Analysis of blood samples from Madeira's patients identified the circulating virus as belonging to dengue serotype 1 (DENV1) with strong sequence similarity to genotypes circulating in Venezuela, Brazil and Columbia at the time [9], [11], [12].

The reporting of short transmission chains of dengue autochthonous cases in European countries is a recent and increasingly common phenomenon [13]–[15]. This first ever dengue outbreak was therefore a sudden event with wide-ranging public health and economic implications, both locally and at the European level. To date, however, neither the conditions that have facilitated this short epidemic and its extinction nor the associated potential for future outbreaks have been studied in detail. Here, we develop an ento-epidemiological mathematical framework to explore the ecological conditions and human-mosquito transmission dynamics underlying this outbreak. Our results indicate that the declining temperatures of autumn were the determining factor for the outbreak's sudden decline. We further estimate that the probable time of introduction was around the end of August, weeks before the first clinical cases were officially reported. Importantly, while this matched with the period when airline traffic (to and from the island) was at its yearly maximum, introductions at an earlier timepoint could have resulted in significantly bigger and longer-lasting epidemics, with obvious consequences for local public health and disease spread to other European countries.

## Materials and Methods

### Ento-Epidemiological Framework

We devised an ordinary differential equation (ODE) model to capture the transmission dynamics of dengue between human and mosquito hosts. The human population is assumed to have constant size (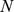) and to be fully susceptible to the virus. Upon challenge with infectious mosquito bites (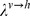), individuals enter the incubation phase (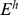) with mean duration of 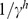 days, later becoming infectious (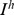) for 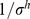 days and finally recovering (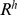) with life-long immunity. The dynamics of the human population are defined by the following set of ODEs:
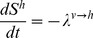
(1)

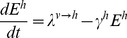
(2)

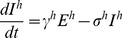
(3)

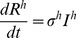
(4)

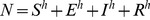
(5)


For the dynamics of the vector population we consider the model previously formulated by Yang and colleagues [16], in which individuals are divided into two pertinent life-stages: aquatic (eggs, larvae and pupae, 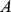) and adult females (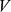). We further extend the adult class by subdividing into the epidemiologically relevant stages for dengue transmission: susceptible (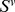), incubating (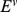) for 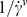 days and infectious (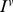). For ease of reading, the temperature-dependent entomological factors are herein distinguished by a  (dot) notation (further details in the following sections). The system of equations describing the vector population is:
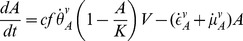
(6)

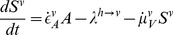
(7)

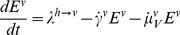
(8)

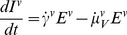
(9)

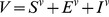
(10)


Here, the coefficients 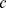 and 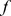 are the fraction of eggs hatching to larvae and the fraction of female mosquitoes hatched from all eggs, respectively. For simplicity and lack of quantifications for the local mosquito population, we assume these to be 1 (see the original publication for a discussion [16]). Moreover, 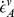 denotes the rate of transition from aquatic to adults, 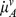 and 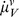 are the mortality rates, and 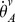 is the intrinsic oviposition rate. The logistic term 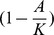 can be understood as the physical/ecological available capacity to receive eggs, scaled by the carrying capacity term 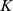, used in the fitting approach to indirectly estimate the adult mosquito population size (see below). From the above system, the basic offspring number (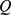), that is, the mean number of viable female offspring produced by one female adult during its entire time of survival (and in the absence of any density-dependent regulation), can be derived as:
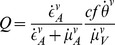
(11)


All parameters defining 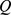 are temperature-dependent (see below). For a fixed temperature 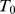 it is possible to derive expressions for the expected population sizes of each mosquito life-stage modelled. These are used to initialize the system, given the temperature present at the initial timepoint:
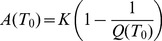
(12)

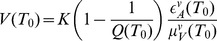
(13)


The vector-to-human (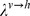) and human-to-vector (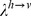) incidence rates are assumed to be density-dependent and frequency-dependent (respectively), in respect to the type of infected host being considered:
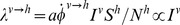
(14)

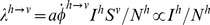
(15)


Here, 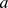 is the biting rate and 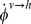 and 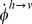 are the vector-to-human and human-to-vector transmission probabilities per bite. This approach follows the recent framework from Althouse et al. which conforms to the expectations arising from the constant nature of the number of bites per mosquito [17]: conceptually, (i) an increase in the density of infectious vectors should directly raise the risk of infection to a single human; while (ii) an increase in the frequency of infected humans raises the risk of infection to a mosquito biting at a fixed rate.

With the two hosts, the expression for dengue's basic reproductive number is defined equally to previous modelling approaches [18], [19] but without human mortality:
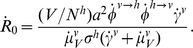



### Temperature-Dependent Parameters

In this section we summarize the methodologies used for each of the seven entomological parameters dependent on temperature (Table 1). Here, 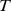 is temperature in Celsius, 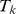 is temperature in Kelvin and 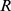 is the universal gas constant in 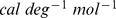.

**Table 1:** Temperature-dependent parameters.

notation	description	reference
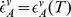	transition rate from aquatic to adult mosquito life-stages	[16]
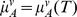	mortality rate of aquatic mosquito life-stages	[16]
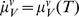	mortality rate of adult mosquito life-stage	[16]
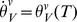	intrinsic oviposition rate of adult mosquito life-stage	[16]
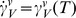	extrinsic incubation period of adult mosquito life-stage	[20]
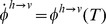	human-to-vector probability of transmission per infectious bite	[23]
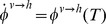	vector-to-human probability of transmission per infectious bite	[23]

Analytical solutions from other studies are used. See Methods section.

**Figure 3:**
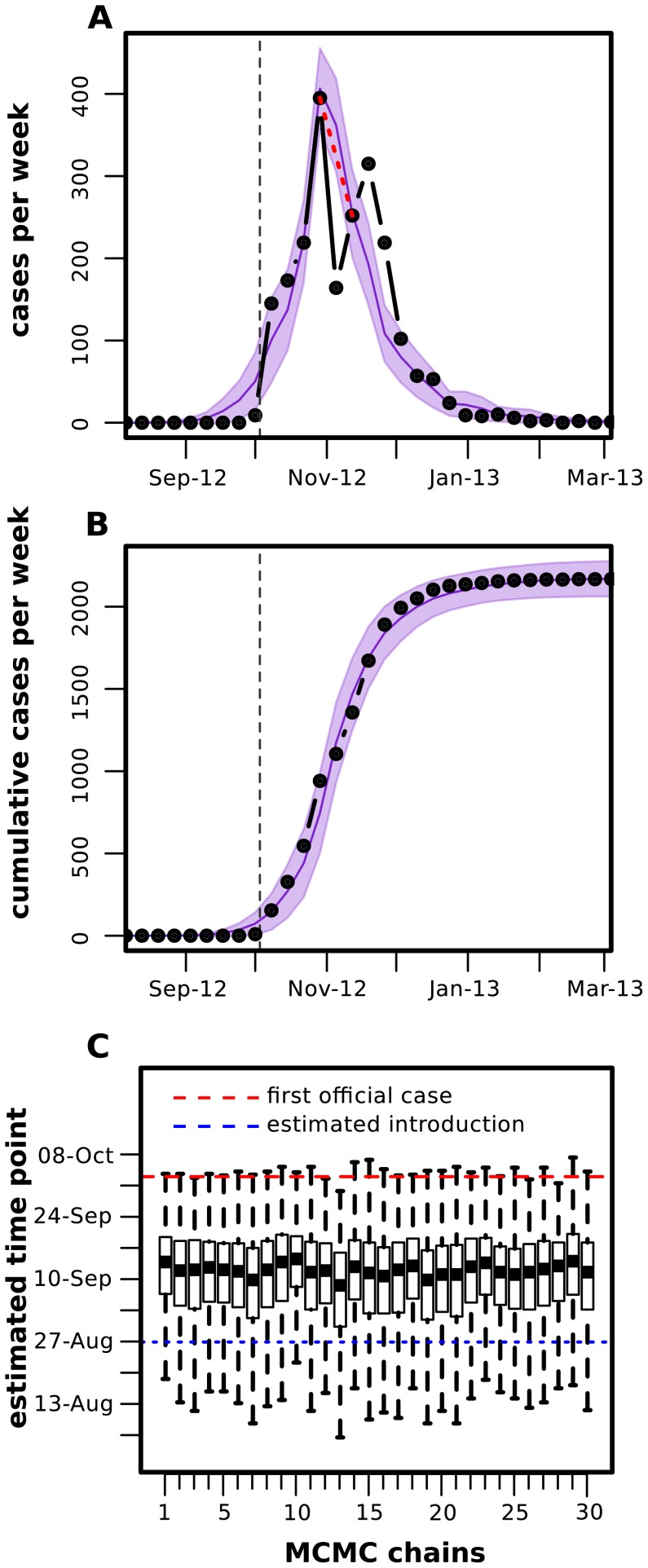
Model fitting to Madeira's dengue outbreak data. (*A,B*) Reported cases (incidence and cumulative) per week (dotted, black) and example of model fitting (solid, purple). Coloured area (purple) is the standard deviation of all accepted steps in the MCMC chain. The dashed vertical line represents the date of the first reported clinical cases. The red dashed line represents the epidemic progression ignoring the first week in November, when a new surveillance method was introduced. (*C*) Stationary distributions of the estimated timepoint of first case for 30 independent MCMC runs with random initial conditions and 1 million steps.

**Figure 4:**
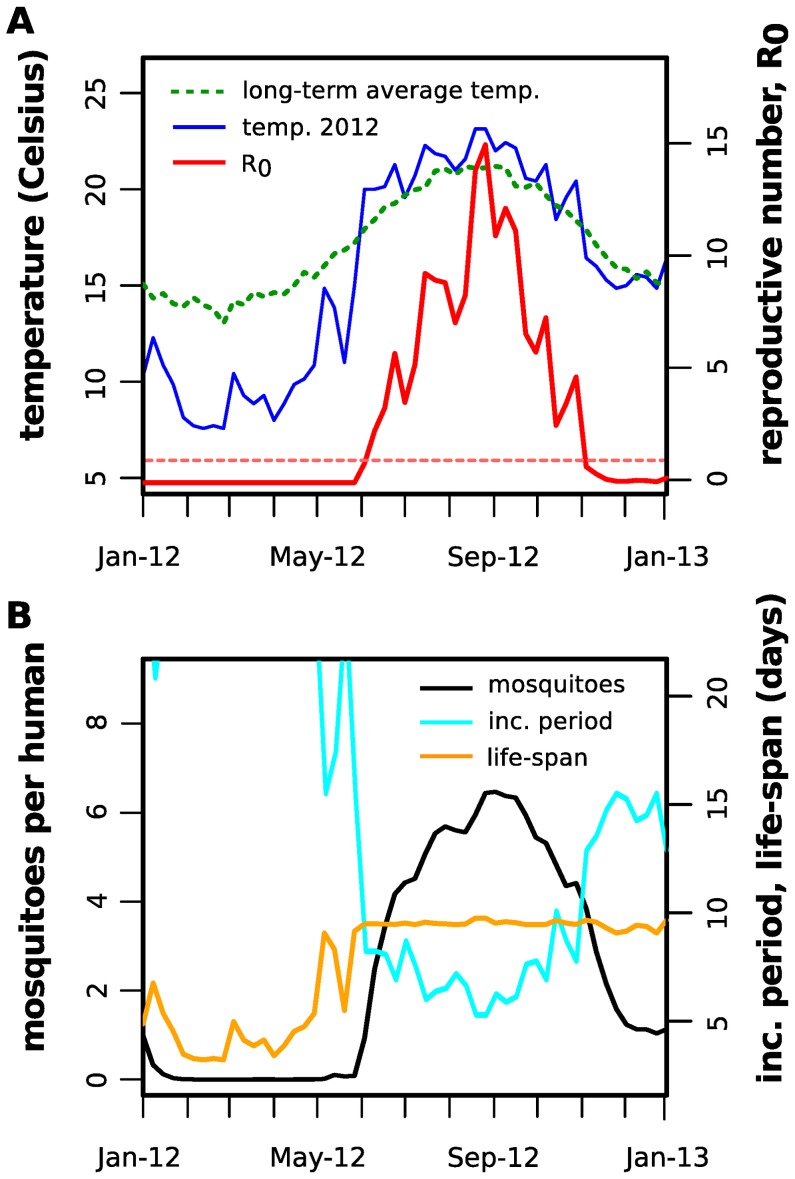
Model-derived epidemiological and entomological parameter estimates for 2012. (*A*) Example of estimated 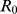 values for 2012 (solid, red) together with the weekly minimum temperatures for 2012 (solid, blue) and long-term average of minimum temperatures (2001–2011, dashed green). The dashed red line marks the epidemic threshold 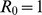. (*B*) Example of estimated number of mosquitoes per human (solid, black), incubation period (solid, cyan) and adult life-span (solid, orange) for 2012.

**Figure 5:**
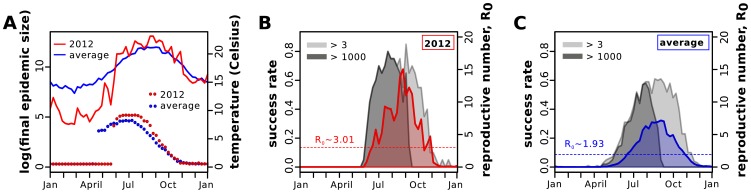
Model-derived epidemic potential for the island of Madeira. (*A*) Temperatures for the year of 2012 (red, solid line) and average temperatures for the past 10 years (2001–2011; blue, solid line). The points mark the mean outbreak size (number of cases) for 100 stochastic introductions at different timepoints using temperature data from 2012 (red) and the average over the past 10 years (blue). (*B*) Derived real-time 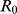 (red, solid line) for 2012, with an annual mean of 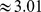 (dashed line). (*C*) Derived real-time 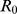 (blue, solid line) for the past 10 year, with an annual mean of 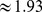 (dashed line). (*B,C*) Grey shaded areas are the frequency of simulations (in 100) achieving either more than 3 (light grey) or 1000 (dark grey) cases.

In the study by Yang et al., from where we base the developmental part of our vector dynamical system (see above), temperature-controlled experiments were performed on populations of *Aedes aegypti* to derive closed-form expressions (based on polynomials) for the model's rates (see Figures 2, 3, 4 and 5 of the original publication [16]). We integrate such solutions into our framework:
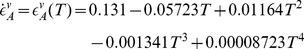


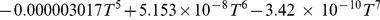
(16)

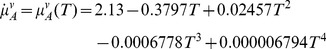
(17)

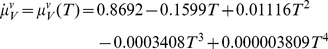
(18)

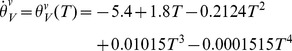
(19)


The relationship between the extrinsic incubation period and temperature has been formulated by Focks et al. [20] using an enzyme kinetics model previously proposed by other authors [21] and used in other dengue modelling approaches [22]. The model assumes that the rate of development is determined by a single rate-controlling enzyme. The expression used is:
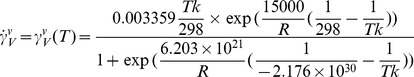
(20)


The probabilities of transmission per mosquito bite 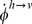 and 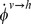 are modelled as previously estimated by Lambrechts and colleagues [23]. The data used in their study was both sampled from several other studies and obtained from *de novo* experiments that measured the variations in proportion of infected and transmitting vectors according to changes in temperature. The analysis was done for a variety of arboviruses from the flavivirus family, including the Dengue virus, the West Nile virus, Murray Valley Encephalitis virus and St. Louis Encephalitis virus. The expressions used are:
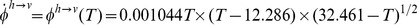
(21)

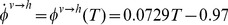
(22)


### Constant Parameters

The framework described above has only three fixed parameters that are neither temperature-dependent nor estimated in the MCMC approach. These can be found in Table 2.

**Table 2:** Constant parameters.

notation	value	description	references
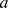	0.4 per day	mosquito biting rate	[44], [45]
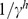	2 days	human latency period	[38], [48]
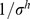	4 days	human infectious period	[49], [50]
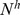	270.000	human population size (Madeira's)	

### Data Series

The outbreak time series was compiled from the official weekly reports from the Direcção Geral de Saúde (Portuguese health ministry) [10] issued throughout 2012 and 2013 and the special report by the European Centre for Disease Prevention and Control (ECDC) [9]. Temperature data for the island of Madeira was assembled from Weather Underground, a Weather Channel's repository [24]. For this we chose a weather station located in the centre of Funchal, Madeira's capital city, where most cases took place. We resorted to the website of Aeroportos da Madeira (Madeira Airports) for the statistics on airline traffic [25]. Finally, the figures for yearly investment in tourism were obtained from the official local source, the Instituto de Desenvolvimento Regional (Institute for Regional Development) [26].

### Markov Chain Monte Carlo Fitting

For the fitting process a Markov chain Monte Carlo approach [27] is used to find combinations of parameters that can describe qualitative properties of Madeira's outbreak. We define the jumping distribution as being symmetric (Gaussian), effectively defining a random walk Metropolis-Hastings algorithm:
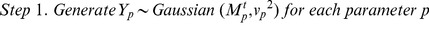
(23)

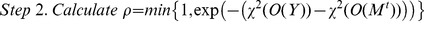
(24)

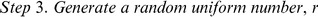
(25)

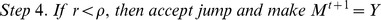
(26)

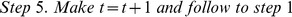
(27)


Here, the Markov chain state is generally denoted by M, the proposal of new parameters by Y and the ODE system (described above) output by O. In step 1, 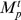 is the Markov chain state of parameter 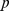 at step 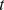, 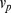 the pre-defined variance for each jump of parameter 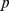 and 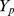 the resulting proposal for time 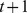. In step 2, 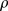 is the probability of acceptance. For this, we calculate the least squares distance between the data series and the ODE output for both the proposal of parameters 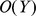 and the previously accepted parameters 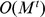. The probability is assumed to decrease exponentially with increases in least squares distances to the data.

With this simple approach we explored all possible combinations of values from four *open* parameters (Table 3) that are able to closely describe the outbreak time series. Amongst these is the carrying capacity K, which we explore in order to indirectly estimate the number of adult mosquitoes per human, and 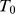, the timepoint of the first case. We also consider two linear coefficients, 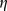 and 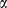, that scale the mortality rate and incubation period of adult mosquitoes - we argue that these entomological factors, as defined by Yang et al. in laboratory experiments [16], should be adjusted to possible biological/ecological local effects. For example, it has been previously demonstrated that mosquito and virus genotype can have an effect on both susceptibility and incubation [28], [29], while human and predator behaviour, as well as the local geospatial topology can affect adult mortality [30], [31]. By considering these linear effects, we do not change the relative effect of temperature variation on mortality and incubation *per se*, but rather allow the baselines to be different from the ones obtained from the laboratory, ideal conditions of Yang et al. study. For a discussion on how much field and laboratory entomological factors can differ, see the recent work by Brady and colleagues [32].

**Table 3:** Estimated parameters.

notation	description	ranges
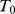	timepoint of first case	(0, date of first empirical case]
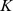	aquatic carrying capacity	(0, 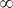 )
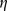	multiplicative (linear) factor for mosquito adult mortality	(0, 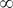 )
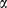	multiplicative (linear) factor for mosquito incubation period	(0, 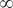 )

Free parameters used by the MCMC approach to fit the data.

We address MCMC convergence by visual inspection and also quantify it using 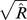, the Gelman-Rubin statistic, which compares the variance between and within M independent MCMC chains [33]. Consider that each chain has length N steps and that 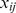 is the 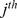 parameter value in chain 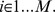 Then, when defining B as the between-chain and W as the within-chain variances, 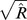 can be obtained using:
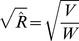
(28)

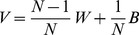
(29)

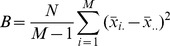
(30)

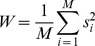
(31)

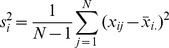
(32)

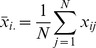
(33)

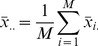
(34)



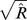
 is expected to approximate 1 when the M chains have converged to the same stationary distribution. Values significantly larger than 1, for instance, indicate that the between-chain variance is greater that the within-chain variance, highlighting that the MCMC may need more time to converge or tuning of jump parameters is required [33]. In our approach we calculate and present 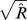 for each estimated parameter (Table 3) using 30 independent chains started with random initial conditions. Our jump parameters are chosen to assure that all MCMC chains presented in this study have acceptance rates 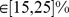 and chains are run for at least 1 million steps.

### Stochastic Formulation

A stochastic version of the ento-epidemiological framework was developed by introducing demographic stochasticity in the transitions of the dynamical system. We used multinomial distributions to sample the effective number of individuals transitioning between classes per time step. Multinomial distributions are generalized binomials, here defined as 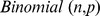, where 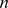 equals the number of individuals in each class and 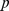 equals the probability of the transition event (equal to the deterministic transition rate). This approach has been demonstrated elsewhere, see e.g. [34].

## Results

To model the introduction of dengue serotype 1 virus into Madeira and investigate the potential for further outbreaks and the island's suitability for dengue endemicity, we developed a mathematical framework for the transmission and population dynamics of dengue and its human and vector hosts (see Methods). The 2012 dengue outbreak presented a sharp initial rise in the number of reported cases and an equally fast decline towards the end of the epidemic (Figure 2). We first considered possible correlations with two known climatic drivers of dengue: temperature and rainfall [35]–[37]. Figure 2 shows the weekly temperature and precipitation data for Funchal, together with the number of reported dengue cases for the period August 2012 - March 2013. We used climate data for Funchal, given the city's predominant role in this outbreak with approximately 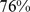 of the total cases up until the second week of November (when the epidemic was already in fast decline) [9].

### Observations and Model Fitting

The rainfall data displays three distinct peaks during this period, a small one coinciding with the start of the outbreak, one during its peak and another two months after the reported cases dropped close to zero (Figure 2). We first note that the actual amount of rainfall over the whole 6-months period under consideration was very small, and it is reasonable to question how much of an impact this could have made on the local mosquito population during the epidemic, especially given the year-round availability of breeding sites [5]. There are also contrasting observations in relationship to rainfall timing and case-response: the first peak was short and small but followed by a drastic increase in case numbers; in contrast, the second much longer and heavier rain episode coincided with the epidemic peak but was followed by a sudden decrease in case numbers; finally, the third peak in rainfall took place outside the time-range of interest. We therefore argued that rainfall was unlikely to have been a main player in the overall progression of this particular short-lived epidemic, although a small contribution cannot be ruled out.

Compared to rainfall, which mostly affects *Aedes's* habitat-quality and availability, temperature directly affects both the mosquito life-cycle and viral replication rates within the mosquito [16], [20], [23], [32], [38]. Accordingly, we note that the drop in minimum temperature towards the end of the year correlated with the decrease in case numbers (Figure 2), possibly delayed by the integrated length of the intrinsic cycles of human-vector-human transmission and aquatic-to-adult mosquito development.

We initially tested a variety of SIR-based model frameworks with constant parameters in time, but it became clear that these models were unable to fit the sudden decline in case numbers after the epidemic peak in November. That is, models that would match the steep exponential rise in incidence, for example, and which would therefore predict very high reproductive numbers (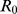), would inevitably generate epidemics of significantly higher magnitude and longer duration than the 2012 outbreak. As human intervention can be ruled out [9], and given the apparent strong correlation between declining temperatures and fading case numbers, we instead focused on a temperature-driven human-mosquito transmission model (see Methods) to gain more insight into the dynamic progression of this outbreak.

In order to fit our ento-epidemiological model to the case data under temperature variations, or more specifically to derive particular parameter combinations that are able to reproduce the timing, shape and size of the epidemic, we employed a Markov chain Monte Carlo (MCMC) approach (see Methods). Figures 3A and B show the fitted model together with the weekly and cumulative incidence data, respectively. There is an overall close fit between model output and the data, although we notice two apparent deviations: one at the onset of the epidemic and one just after its peak. We argue that these are more likely artefacts of the data, however, rather than real discrepancies. That is, the sudden drop in incidence after the epidemic peak is likely due to the introduction of a new surveillance system in the first week of November [9], whereas the slight overestimation of cases during the initial phase of the outbreak could be due to the deterministic nature of our model and possible under-reporting at the onset of the epidemic. We also note that the new surveillance system did not change the clinical or laboratory-confirmation definitions *per se* (see annexe of [9] for case definition) but aimed at efficiently integrating data from all health care centres involved, including the private and public sectors. It is therefore not expected that this change in the system affected the sensitivity of case detection but instead increased the time and space resolution of the epidemic data from November onwards.

Using this method we estimated that the possible timepoint of introduction of dengue to the island occurred towards the end of August, with the first autochthonous human infection between the 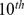 and 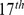 of September, two to three weeks before the first reported clinical case. Convergence to this date (range) was confirmed by independent MCMC runs as demonstrated in Figure 3C. That is, given random initial conditions for the four free parameters (Table 3), the system robustly converged towards equally distributed parameter estimates (by design, this approach produces parameter distributions rather than point estimates); see Methods and Supplementary Figures S1 and S2 for the resulting distributions of other parameters and quantification of convergence.

At the time of the first local human infection, temperatures were already on a declining trajectory (Figure 2), which caused a significant reduction in the virus's reproductive potential due to a combination of shorter mosquito life-expectancy, smaller population size and an increase in the extrinsic incubation period. These effects are demonstrated in Figure 4A, which also illustrates the colder than usual winter and slightly warmer summer during 2012 (compared to the average temperatures over the past 10 years). Dengue's estimated reproductive potential, 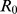, is here given as a time-dependent quantity to highlight the temperature-driven dependencies of entomological factors. It is important to note that this differs from the often used time-varying *effective* reproduction number, 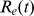, that takes into account the varying susceptibility levels in the population (see Methods for mathematical expressions).

Of particular interest is the increase in the length of the extrinsic incubation period beyond the mosquitoes' average life-expectancies (Figure 4B), substantially contributing to the sharp drop in 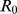 from 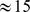 at its peak at the end of the summer, to 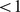 during late autumn and winter. We thus believe that this temperature-driven phenomenon might explain the rapid decrease in dengue incidence and essentially the end of the outbreak, with the expected delay due to the total length of the transmission and developmental cycles.

### Epidemic and Endemic Potential

Using these parameter insights we next investigated potential outcomes if the pathogen would have been introduced at different timepoints during 2012 and further considered introductory events during a ‘typical’ year using average temperatures of the past 10 years (2001–2011). In order to take into consideration the probabilistic nature of viral introduction and epidemic outcome, we expanded our framework to include demographic stochasticity and viral extinction (see Methods section).

As demonstrated in Figure 4B, there was a time window of several months during 2012 when adult mosquito counts where sufficiently high and, critically, the virus's temperature-dependent extrinsic incubation period was shorter than the mosquito's average life-expectancy, thus allowing for efficient vector-human transmission. When simulating introduction events, we found significant differences in the *epidemic windows* between 2012 and a typical year due to deviations in temperature trends throughout the studied periods. Notably, the winter in 2012 was unusually cold, which resulted in a shorter window during which outbreaks could be measured (Figure 5A). At the same time, slightly warmer temperatures during the summer months of 2012 increased the transmission potential, 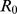 (Figures 5B and 5C), and resulted in bigger outbreaks when compared to a typical year (Figure 5A).

At first sight, differences of 2–4° Celsius in the summer months may seem insufficient to explain the differences in 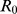 and consequently in outbreak sizes. However, according to experimental evidence, increases in temperature just above the critical point of 20° Celsius will strongly add to the overall vectorial capacity of *Aedes* mosquitoes [16], [20], [23], [32], [38]. This is a consequence of slight changes in the rates describing mortality, incubation and life-stage progression, which in concert have a cumulative effect and may be involved in positive feedback relationships. Hence, differences of a few degrees, especially when maintained over wide periods of time, can have significant and long lasting effects on the vector population and therefore on dengue's reproductive number.

We further investigated dengue's success of invasion into the island by quantifying the frequency of stochastic simulations that developed into outbreaks above certain sizes, differentiating between 2012 (Figure 5B) and ‘typical’ years (Figure 5C). Comparing the occurrence of any-size (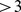) or major-size (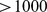) outbreaks, we found the risk for the latter to be strongly linked to introductions during the summer months. In fact, as demonstrated in Figures 5B and 5C, there is a substantial risk for major epidemic outbreaks for introductory events taking place weeks or even months before 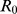 reaches its full potential. This is because any introduction during that period can enjoy the climate-driven ‘deterministic’ growth in 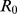 until late summer. We can thus identify a key *epidemic window*, dictated by temperatures above 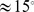 Celsius, in which efforts to detect and control imported cases are crucial for public health planning in Madeira.

In agreement with the estimated differences in transmission potential between 2012 and average years, we also found the invasion success to be generally higher during 2012, which could potentially explain the success of the virus in that particular year. In fact, our results suggest that during a typical year a substantial proportion of introductions (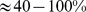) are expected to go extinct before reaching epidemic potential, even during the peak in transmission potential (Figure 5C). Given the homogeneous assumptions of our modelling approach, we argue that these rates should be seen as ‘best’ case scenarios for the successful invasion of dengue in Madeira. In a more realistic scenario, in which heterogeneities in contacts and host and vector densities are present, we expect these rates to be potentially much lower, which could offer an explanation as to why dengue had failed to achieve sustained transmission on the island in the past.

## Discussion

The 2012 dengue epidemic in Madeira was the first European outbreak showing significant and prolonged autochthonous transmission. With *Aedes aegypti* firmly established on the island and travel patterns in place connecting Madeira with other African and South American countries where dengue is now endemic or epidemic, it can be argued that introduction and sustained transmission was only a matter of time. Here, we used a mathematical modelling approach to investigate the underlying drivers of this important epidemic and to highlight the risks of potential future outbreaks.

Of particular importance was the date when the virus had been introduced to the island together with the prevailing ecological conditions at this point and the months that followed. Whereas the first official clinical cases were reported on 3 October 2012, our method dates the timepoint of introduction just over a month earlier, at the end of August. There are various reasons for this discrepancy. Firstly, there is evidence that these initial two cases were the result of autochthonous transmission, i.e. they were not the individuals who introduced the virus to the island but rather subsequent cases [9]. Secondly, dengue infections are frequently asymptomatic [2], which means that several people could have been infected before some individuals developed symptoms sufficiently severe and/or specific for health care officials to suspect for dengue fever. Together, this could have led to a significant under-reporting, a common feature of dengue-endemic regions [2], [39], [40], especially during the onset of the epidemic. These initial cases were followed by a rapid rise in dengue incidence over the following month, with the epidemic peaking around early November, indicative of a high transmission potential at this point of the year. Our estimates of 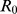, however, showed that its maximum had been reached in August, a few months before the outbreak took place.

To further investigate the causes and dynamics of this epidemic we addressed the conditions on the island during the relevant period. We looked for the possible role of local temperature variation and rainfall. We argued that the timing and strength of the three observed rain episodes was insufficient to have played a critical role in the outbreak, especially as the actual amount of precipitation was very small in each of these episodes. Furthermore, it is plausible that the year-round availability of breeding sites in flower and plant pots, as previously described in entomological studies of Madeira [5], [9], [41], may reduce the impact of short and sporadic rain episodes by allowing the mosquito population to persist throughout the year. Temperature, on the other hand, due to its aforementioned influence on the extrinsic incubation period, adult mortality and aquatic developmental rates, appeared to be the predominant driver and essentially limiting factor of the 2012 outbreak. According to our model, the temperatures in autumn not only caused a reduction in the number of adult mosquitoes but, crucially, dropped bellow the critical threshold where the incubation period is shorter than the average mosquito life-span and onward transmission to humans becomes probable. This effectively reduced vectorial capacity and stopped viral propagation, causing a significant decrease in dengue cases.

Given the natural annual variation in temperature on the island we found significant differences in dengue's transmission potential between summer and winter months. This is a consequence not only of varying mosquito population sizes but also of other temperature-dependent entomological and viral factors. During the warmer months, 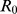 could reach 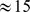 for a few weeks, which stands just above the often reported range of 2–12 for dengue [42]. However, the estimates present in the literature are often based on methods that necessarily average the transmission potential over long periods of time, such as months, transmission seasons or, more commonly, years. In contrast, our estimates of 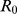 are point estimates that follow temperature variations in real time. Crucially, when averaged over 2012, we obtained values of 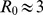, in line with estimates from age-stratified sero-prevalence studies [43]. This, on the other hand, highlights some of the dangers in determining dengue's region-specific 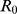 based on averages over long periods of time, as the true values might vary significantly within just a couple of weeks due to temperature oscillations (as demonstrated here and in [16], [20], [23]) but also due to the heterogeneous and volatile nature of mosquito populations [6], [30], [44], [45].

Using the parameter estimates from fitting our model to the 2012 outbreak data, we simulated other scenarios where the virus was introduced at different timepoints during the year. For this, we separately considered temperature data for 2012 and the average for the past 10 years (2001–2011). The latter was used to make predictions on the epidemic and endemic potential of dengue during an average year in Madeira. Due to the slightly warmer summer in 2012 we found both the epidemic potential and probability of invasion to be higher when compared to a typical year on the island, potentially explaining the success of the virus in that particular year. However, our results also indicated a reasonable invasion potential between late April and October based on average temperatures and thus identified a key epidemic window, during which efforts by the local authorities should take place to prevent importation, to control the mosquito population and to raise awareness of residents, specially in Funchal. Importantly, there is little support for dengue to become endemic in Madeira, since temperatures regularly drop bellow 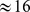 Celsius outside this window, which severely affects several entomological and viral factors and effectively reduces vectorial capacity to unsustainable levels.

Our results can also be used to discuss the potential implications for spreading dengue from the island to other countries. As mentioned in the Introduction and shown in Figures 1B–D, Madeira has a high influx of visitors, mostly from other European countries as well as South America. These are concentrated around two distinct holiday peaks, one in Easter and one during the main summer holiday season in August / September (Supplementary Figure S3). While our estimated timepoint of introduction of dengue coincided with the height in tourism around the end of August, which might explain the dynamics of the events that followed, it is important to note that the outbreak reached its peak when average tourist numbers had already dropped to their annual minimum (Figures 2 and S3), thus limiting the potential for disease exportation. Even so, a total of 81 reported cases were exported to European cities, a number that is possibly underestimated due to asymptomatic dengue infections [2]. This clearly demonstrates the future potential for spreading dengue from the island to continental European areas, with a particularly high risk for those regions with warm climates and where *Aedes albopictus* is well established, such as Italy or Southern France (Supplementary Figure S4).

Some caution must be urged about the interpretation of some of our predictions. Our modelling approach was designed to address the qualitative relationships between viral, human and entomological factors that may have dictated the success and demise of Madeira's dengue outbreak. However, this dynamic framework includes key assumptions that may affect estimations such as epidemic sizes and invasion success. For instance, recent modelling work suggests that spatial segregation between dengue's hosts greatly reduces the propensity for large-scale outbreaks by restricting the pathogen's access to the susceptible pool [19]. It is also known that demographic stochasticity plays an important, if not crucial role for the transmission of human pathogens [46], including dengue virus [19], [47]. We have made an effort to account for this by investigating the epidemic and endemic potential of Madeira using a stochastic version of our model. However, in order to keep the MCMC fitting methodology simple and robust, our parameter estimations were still dependent on deterministic assumptions, and the quantifications of invasion success and epidemic potential should thus be understood as average, if not worst-case scenarios. On the other hand, using this MCMC fitting approach allowed us to capture some of the expected underlying uncertainty, for example with regards to the possible timepoint of introduction (Figure 3C), despite using an underlying deterministic framework.

In summary, we have shown that the 2012 dengue outbreak in Madeira was predominantly self-limited, driven to extinction by falling temperatures rather than human intervention. Our results demonstrate that there is little that supports the possibility of dengue to become endemic on the island; there is, however, a major risk for future epidemic outbreaks, with their likelihood significantly enhanced during periods of increased travel from dengue-endemic countries. These outbreaks are only expected within a limited window of time between late spring and summer. Control and social awareness efforts should therefore be placed within this time window to reduce economic and public health consequences, not only for Madeira but also for other European countries with strong tourism links to this island.

## Supporting Information

Figure S1
**Markov chain Monte Carlo stationary distributions.** (*A*–*D*) Stationary distributions for 30 independent MCMC runs with random initial conditions and 1 million steps. For quantification of convergence, see Supplementary Figure S3. (*A*) The timepoint of introduction, T0; (*B*) the aquatic carrying capacity factor, K; (*C*) the linear factor scaling the adult mosquito incubation period, *α*; and (*D*) the linear factor scaling the adult mosquito mortality rate, 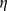.(PDF)

Figure S2 **Markov chain Monte Carlo convergence quantification.** Gelman-Rubin statistic for the 30 independent MCMC runs started with random initial conditions as in Supplementary Figure S1. Convergence is detected, as the Gelman-Rubin statistic closely approximates 1 for all estimated parameters.
(PDF)

Figure S3 **Average airline passengers entering Madeira per month.** Data averaged for the period between 1991 and 2012.
(PDF)

Figure S4 **Known distribution of** ***Aedes albopictus*** **in Europe.** Distribution as updated on October 2013 by the European Centre for Disease Prevention and Control.
(PDF)

Figure S5
**Example of the stochastic dynamics for 2012.** (*A*) Mean dynamic behaviour (lines) of the model for different timepoints of introduction (arrows) using 2012's parameters and temperature-series (see Figures 4 and 5 of the main text). Shaded areas are the standard-deviation. (*B*) Derived real-time 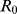 (red, solid line) which is 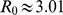 (red, dashed line) when averaged over the year. Grey shaded areas are the frequency of simulations (in 100) achieving either more than 3 (light grey) or 1000 (dark grey) cases.(PDF)
