# Supplementary material for: The 2012 Madeira Dengue Outbreak: Epidemiological Determinants and Future Epidemic Potential
Source: PLoS Negl Trop Dis. 2014 Aug 21;8(8):e3083. doi: 10.1371/journal.pntd.0003083 (PMC4140668; doi:10.1371/journal.pntd.0003083)
Supplement: Figure S1 — Markov chain Monte Carlo stationary distributions. (A–D) Stationary distributions for 30 independent MCMC runs with random initial conditions and 1 million steps. For quantification of convergence, see Supplementary Figure S3. (A) The timepoint of introduction, T0; (B) the aquatic carrying capacity factor, K; (C) the linear factor scaling the adult mosquito incubation period, α; and (D) the linear factor scaling the adult mosquito mortality rate, . (PDF) [file pntd.0003083.s001.pdf]

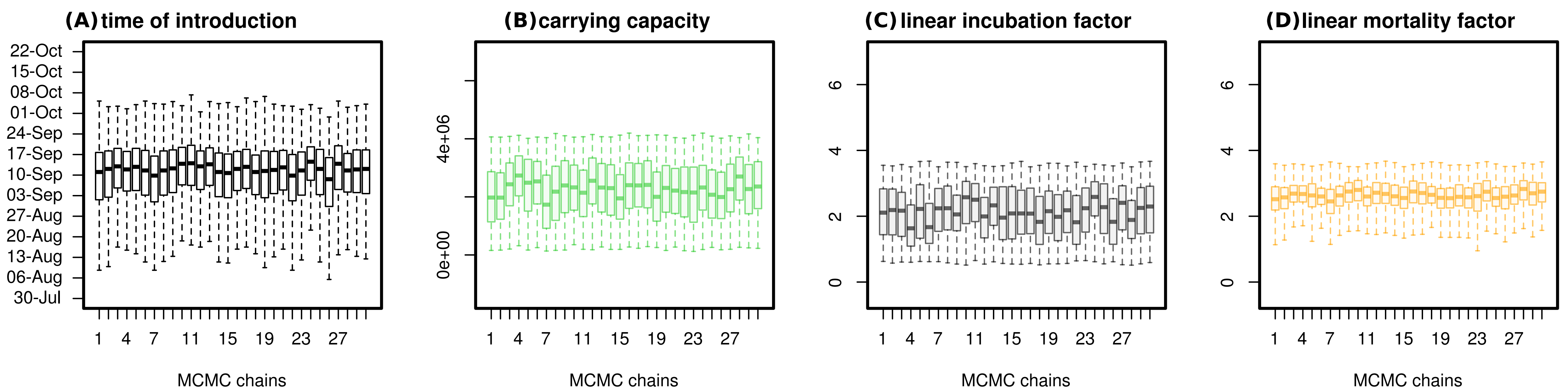

(A) time of introduction
22-Oct
15-Oct
08-Oct
01-Oct
24-Sep
17-Sep
10-Sep
03-Sep
27-Aug
20-Aug
13-Aug
06-Aug
30-Jul
1
4
7
11
15
19
23
27
MCMC chains
(B) carrying capacity
4e+06
0e+00
MCMC chains
(C) linear incubation factor
6
4
2
0
MCMC chains
(D) linear mortality factor
MCMC chains
