# Supplementary figures and images for: The 2012 Madeira Dengue Outbreak: Epidemiological Determinants and Future Epidemic Potential
Source: PLoS Negl Trop Dis. 2014 Aug 21;8(8):e3083. doi: 10.1371/journal.pntd.0003083 (PMC4140668; doi:10.1371/journal.pntd.0003083)

## Gelman-Rubin statistic

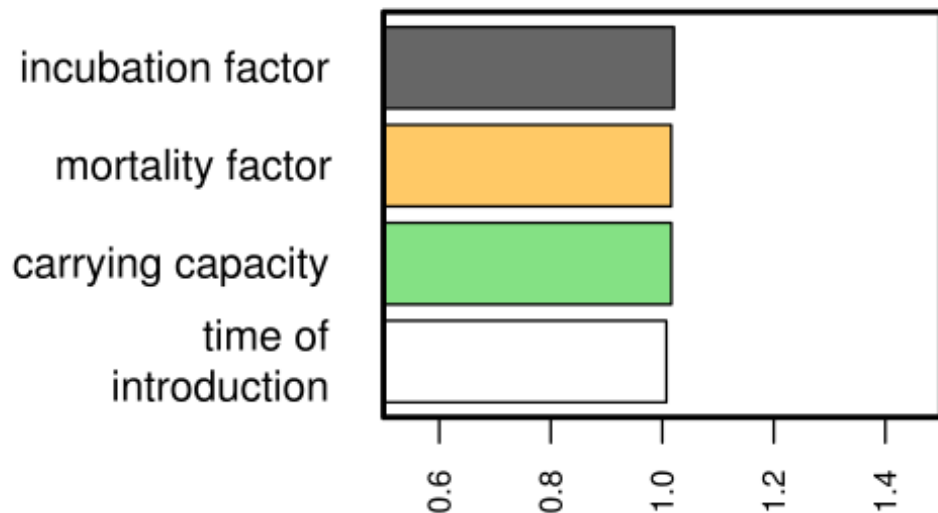

Supplement: Figure S2 — Markov chain Monte Carlo convergence quantification. Gelman-Rubin statistic for the 30 independent MCMC runs started with random initial conditions as in Supplementary Figure S1. Convergence is detected, as the Gelman-Rubin statistic closely approximates 1 for all estimated parameters. (PDF) [file pntd.0003083.s002.pdf]

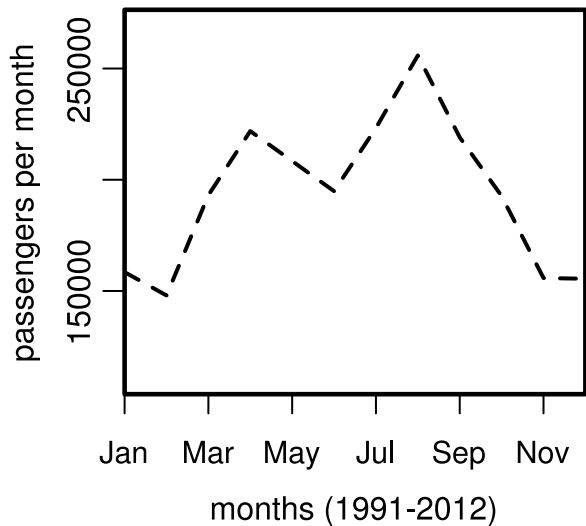

Supplement: Figure S3 — Average airline passengers entering Madeira per month. Data averaged for the period between 1991 and 2012. (PDF) [file pntd.0003083.s003.pdf]

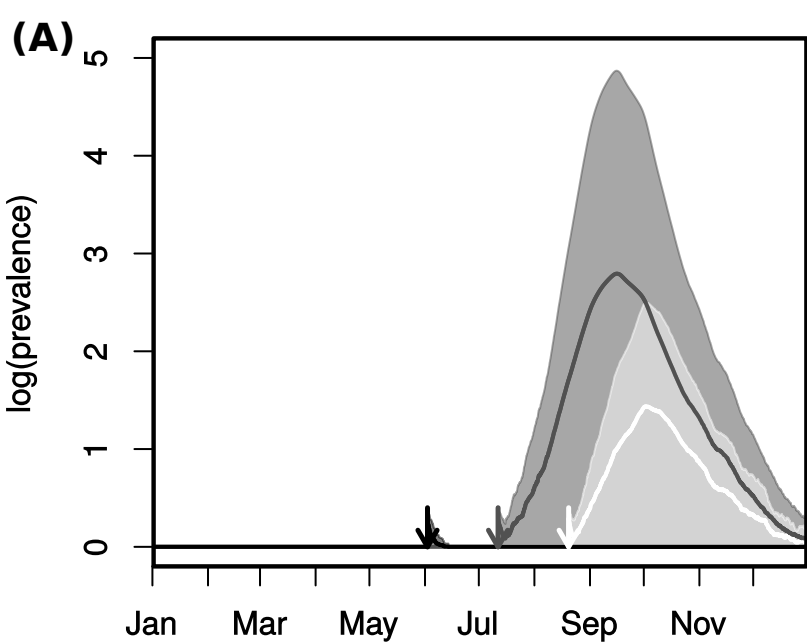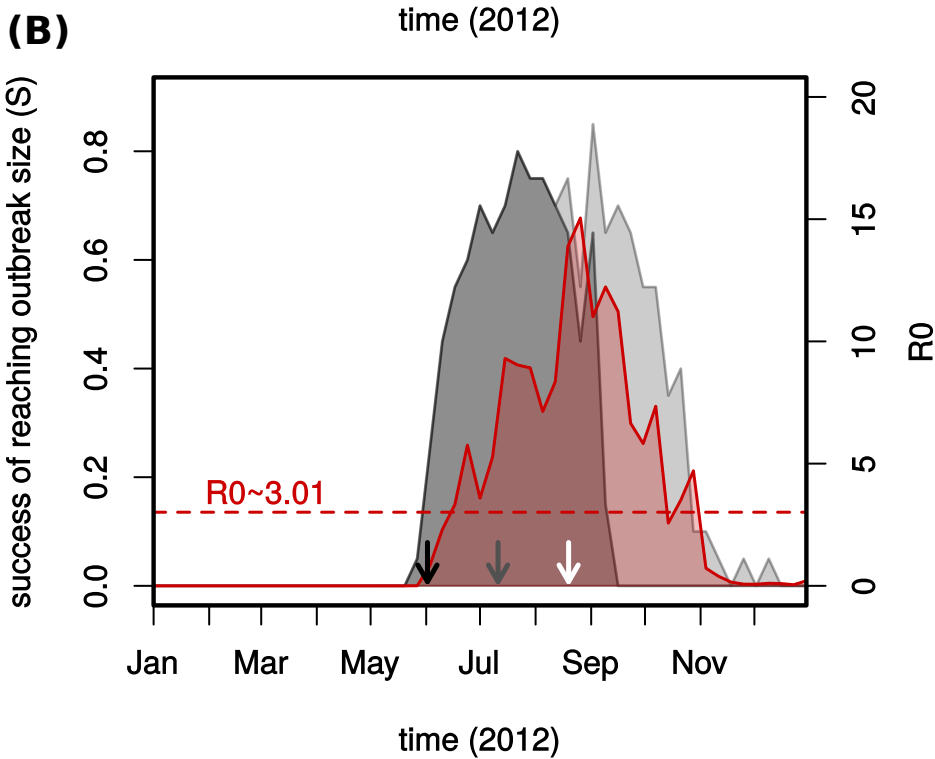

Supplement: Figure S5 — Example of the stochastic dynamics for 2012. (A) Mean dynamic behaviour (lines) of the model for different timepoints of introduction (arrows) using 2012's parameters and temperature-series (see Figures 4 and 5 of the main text). Shaded areas are the standard-deviation. (B) Derived real-time (red, solid line) which is (red, dashed line) when averaged over the year. Grey shaded areas are the frequency of simulations (in 100) achieving either more than 3 (light grey) or 1000 (dark grey) cases. (PDF) [file pntd.0003083.s005.pdf]
