# Supplementary material for: The 2012 Madeira Dengue Outbreak: Epidemiological Determinants and Future Epidemic Potential
Source: PLoS Negl Trop Dis. 2014 Aug 21;8(8):e3083. doi: 10.1371/journal.pntd.0003083 (PMC4140668; doi:10.1371/journal.pntd.0003083)
Supplement: Figure S4 — Known distribution of Aedes albopictus in Europe. Distribution as updated on October 2013 by the European Centre for Disease Prevention and Control. (PDF) [file pntd.0003083.s004.pdf]

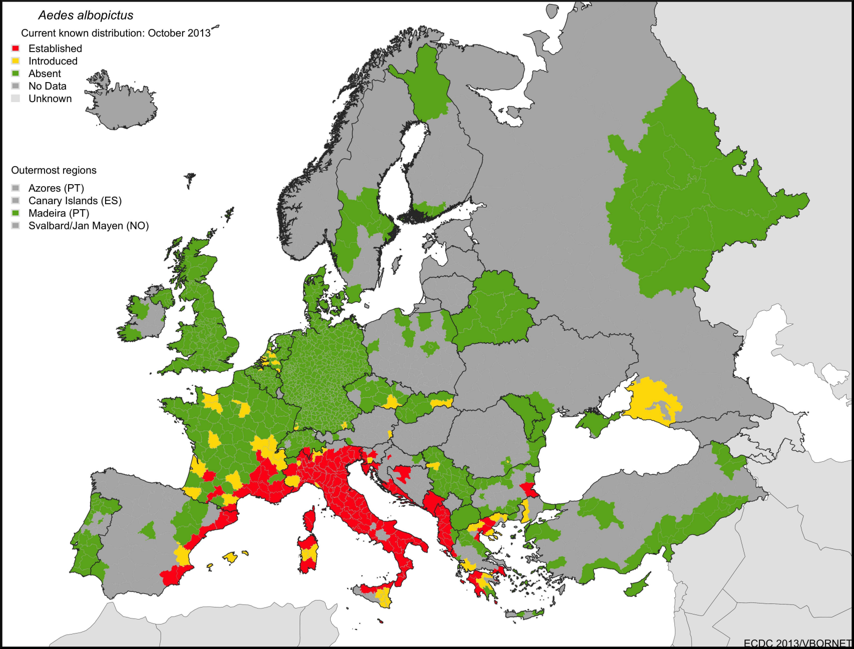

Aedes albopictus
Current known distribution: October 2013
Established
Introduced
Absent
No Data
Unknown
Outermost regions
Azores (PT)
Canary Islands (ES)
Madeira (PT)
Svalbard/Jan Mayen (NO)
ECDC 2013/VBORNET
